# The feasibility study of using multiple partial volumetric‐modulated arcs therapy in early stage left‐sided breast cancer patients

**DOI:** 10.1120/jacmp.v13i5.3806

**Published:** 2012-09-06

**Authors:** Ping‐Fang Tsai, Shih‐Min Lin, Shen‐Hao Lee, Chie‐Yi Yeh, Yi‐Ting Huang, Chung‐Chi Lee, Ji‐Hong Hong

**Affiliations:** ^1^ Department of Radiation Oncology Chang Gung Memorial Hospital Tao‐Yuan Taiwan; ^2^ Department of Medical Imaging and Radiological Science Chang Gung University Tao‐Yuan Taiwan

**Keywords:** RapidArc, VMAT, breast cancer, active breathing control (ABC), deep inspiration breath‐hold (DIBH)

## Abstract

The purpose of this study was to assess the feasibility of using a multiple partial volumetric‐modulated arcs therapy (MP‐VMAT) technique on the left breast irradiation and to evaluate the dosimetry and treatment efficiency. Ten patients with left‐sided breast cancer who had been treated by whole breast irradiation were selected for the treatment plan evaluation by using six partial volumetric modulated arcs. Each arc consisted of a 50° gantry rotation. The planning target volumes and the normal organs, including the right breast, the bilateral lungs, left ventricle, heart, and unspecified tissue, were contoured on the CT images. Dose‐volume histograms were generated and the delivery time for each arc was recorded. The PTV received greater than 95% of the $V_{95}$ for all cases, and the maximum dose was within ±1% of 110% of the prescription dose. The mean homogeneity index (HI) was 10.61±0.99, and mean conformity index (CI) was 1.21±0.03. The mean dose, $V_{5}$, $V_{10}$, $V_{25}$, and $V_{30}$ of the heart were 7.61±1.38 Gy, 59.73% ±15.87%, 24.39% ±6.82%, 2.52% ±1.11%, and 1.57% ±0.71%, respectively. The volume of the left ventricle receiving 25 Gy was 5.15% ±2.23%. The total lung mean dose was 5.57±0.36 Gy, with $V_{5}$ of 25.39% ±3.88% and $V_{20}$ of 5.66% ±0.89%. The right breast received a mean dose of 2.13±0.22 Gy, with $V_{5}$ of 1.83% ±1.22% and $V_{10}$ of 0.04% ±0.12%. The mean dose of unspecified tissue was 5.34±0.37 Gy and $V_{5}$ was 22.23% ±1.57%. The volume of the unspecified tissue receiving 50 Gy was 0.50% ±0.14%. The mean delivery time for each arc was 13.9 seconds. The average MU among ten patients was 511 MU (range 443 to 594 MUs). The MP‐VMAT technique for the left‐sided breast cancer patients achieved adequate target dose coverage while maintaining low doses to organs‐at‐risk, and therefore reduced the potential for induction of second malignancy and side effects. The highly efficient treatment delivery would be beneficial for improving patient throughput, providing patient comfort, and achieving precise treatment with the breathing control system.

PACS number: 87.55.‐x, 87.55.D‐, 87.55.dk

## I. INTRODUCTION

Although partial volume irradiation has been advocated recently, in many centers, whole breast irradiation (WBI) remains the standard treatment for early‐stage breast cancer patients after breast‐conserving surgery. Traditionally, two tangential photon beams with wedge pairs or the field‐in‐field (FIF) technique^(1)^ were employed for WBI to cover the entire breast with enough margins to reduce the irradiated volume of the lung and heart and avoid hot spots inside the treatment field. Although the tangential field technique achieved a good local control rate in breast cancer patients, an increased risk of acute radiation dermatitis,^(2)^ late cardiac,^3^, ^6^ pulmonary toxicity,^7^, ^9^ and secondary breast cancer on the contralateral breast^10^, ^12^ were shown, mainly caused by the inhomogeneous radiation distributed within the breast, the substantial lung and heart volumes still in the high‐dose region, and some doses given to the contralateral breast.

Many advanced techniques, such as fixed‐beam intensity‐modulated radiation therapy (IMRT)^13^, ^15^ and helical tomotherapy,^16^, ^18^ have been used to provide a better dosimetric result. These techniques have been shown to improve dose conformity to the target and minimize the high dose to critical organs.^17^, ^18^ However, common drawbacks of these advanced techniques include the extended time needed, higher monitor units of the beam delivery,^(19)^ and an increased low‐dose bath area.^13^, ^14^, ^17^, ^20^ Volumetric‐modulated arc therapy (VMAT),^(21)^ an improvement of the IMRT technique, was developed in 2008, and was named as RapidArc by Varian Medical Systems. The RapidArc technique is capable of modulating gantry speed, multileaf collimator (MLC) speed, and dose rate while the gantry is in rotation. Compared to fixed‐beam IMRT and helical tomotherapy, the beam delivery time of the VMAT technique was reduced to approximately 1.5 to 3 minutes for a 2 Gy irradiation, and the monitor units of the beam were also decreased.^(21)^ Thus, some recent publications^19^, ^22^ have investigated the potential of using the VMAT technique on whole breast irradiation.

The WBI was often performed during free breathing; the mismatch of dose distribution with patient motion has been compensated for using a skin flash technique. In order to average out the dose near the body outline area, a skin flash has been created by adding a 2 cm field size opening on the conventional techniques or the fluence expansion outside the body contour on the IMRT. Since the breast position might change with respiration, the reproducibility of the dosimetry during the dynamic beam delivery in gantry rotation deserves special attention. A preclinical evaluation of respiratory gating with RapidArc^(23)^was introduced recently. It may prolong the total treatment time in a clinical situation and eventually lose the benefit of the fast delivery time on the RapidArc compared to deep inspiration breath‐hold (DIBH) technique. Since respiratory pattern is not always regular and repeatable during the treatment session, controlling breath movement should be a better way to reduce dosimetric uncertainty. Korreman et al.^(24)^ had shown that voluntary DIBH reduces cardiac doses simultaneous with significant pulmonary tissue sparing, in comparison to the end‐inspiration gating technique. In breast cancer, many investigators^25^, ^31^ have tried to minimize the variation of the breast motion and reduce the heart and lung volume in the irradiation field^25^, ^29^, ^30^ through deep inspiration by various breath‐holding techniques, such as active breathing control (ABC)^26^, ^28^, ^31^ and DIBH. The treatment usually needs to be broken down into a couple of sections while employing these techniques because the beam delivery time is too long to be given in one breath‐holding. To minimize patient discomfort and treatment uncertainties, the development of fast delivery techniques is essential. Unfortunately, this issue has not been fully explored by the literature search.

In this study, we describe a multiple partial volumetric modulated arc therapy (MP‐VMAT) technique for WBI. The goals are to improve the dosimetric results with a highly efficient delivery time so that it is feasible to incorporate the breath‐holding technique to control organ motion during the beam delivery. The dose‐volume histogram (DVH) was calculated and the efficiency of treatment was measured.

## II. MATERIALS AND METHODS

### A. Patient selection

Ten patients with early stage, left‐sided breast cancer treated by conventional technique were selected for this retrospective analysis in dose distribution and treatment efficiency by MP‐VMAT planning. The prescription was a 1.8 Gy daily dose and 50.4 Gy total dose in 28 fractions. The patients were simulated in a supine position with the arm abducted (90° or greater) on the disease side. The computed tomography (CT) images were obtained with a 16‐slice large‐bore CT simulator (GE Healthcare, Salt Lake City, Utah) at 5.0 mm slice spacing, extending from the bottom of the lungs to 5 cm superior to the breast. The data was then transferred to a treatment planning system for targets and critical structures delineation.

### B. Treatment planning

The clinical target volume (CTV), PTV, contralateral (right) breast, contralateral (right) lung, ipsilateral (left) lung, total lung volume (TLV), heart, left ventricle, and unspecified tissue were contoured on the CT images. The CTV was defined as the whole breast volume, excluding the pectoralis muscles, chest wall muscles, ribs, and 5 mm inside the body surface. The PTV was the three‐dimensional expansion of CTV with a 7.0 mm margin in all directions, considering the daily setup variation and the possible intrafraction motion, and 5.0 mm from the body surface was also excluded. The PTV ranged from $397.7\;{\text{cm}}^{3}$ to $756.3\;{\text{cm}}^{3}$ and the mean volume was $562.1\;{\text{cm}}^{3}$. The unspecified tissue structure was defined as the volume of the whole CT images minus all the delineated targets and critical structures. All the targets and critical organ delineation were performed by the same physician, and the contouring rules as described in the Radiation Therapy Oncology Group (RTOG) breast cancer atlas were followed. The heart was contoured from the pulmonary trunk branches into the left and right pulmonary arteries, and to its apex according to the RTOG 0413 protocol. The left ventricle was contoured from the mitral valve at the cephalic direction along the smooth appearance of the left ventricular outflow tract and the posterior border was along the diaphragmatic cardiac surface. The bilateral lungs were generated automatically from the segmentation function in the Eclipse treatment planning system (TPS) (Varian Medical Systems, Palo Alto, CA). The planning organ‐at‐risk volume (PRV) was created to separate the targets and the critical structure for optimization purposes. The heart and left lung PRVs were generated from the original organ volume, excluding 5 mm away from PTV. All the targets, critical organs, and PRV were used for optimization.

Both the treatment planning and RapidArc optimization were performed with version 8.6 of the Eclipse TPS on a Varian iX series linear accelerator equipped with a 120 Millennium MLC with 5 mm leaf width. Six MV photon energy and a maximum dose rate of 600 MU/min were used for the MP‐VMAT plans. The Anisotropic Analytic Algorithm (AAA) with tissue heterogeneous correction and grid size of 0.25 cm were used for dose calculation. The planning dose constrains were developed on the basis of the retrospective analysis of literature reviews and a prerun MP‐VMAT treatment plan. The dose constrains and the relative priorities used during the dynamic arc optimization for this study are listed in Table 1. In version 8.6 of the Eclipse TPS, the dose dynamic arc optimization proceeds through five multiresolution levels (levels 1 to 5). On the last two resolution levels, the ratio of the priorities between the target to the highest priority on the critical organs were increased to 1.6~1.8 in order to further maximize the dose differences between the target and the critical organs for this study. All the treatment plans presented in this paper were optimized once and including one time elimination of hot spots. Currently, there is no skin flash function available for RapidArc in Eclipse TPS version 8.6. The focus of this study is mainly to assess the feasibility of the MP‐VMAT technique and the dosimetric advantages to the critical organs.

**Table 1:** The dose constrains and the relative priority used during optimization for multiple partial VMAT planning.

*Structures*	*Criteria*	*Dose Limit (% of Rx dose)/(Gy)*	*Priority (Level 1–3)*	*Priority (Level 4–5)*
PTV	$V_{95}$	>95% of Rx	120	400
Maximum dose	0%	>110%	250	450
Heart	Mean dose	9Gy	120	120
	$V_{5}$	<70%		
	$V_{10}$	<30%		
	$V_{25}$	<5%		
	$V_{30}$	<2.5%		
Left Lung	Mean dose	10Gy	80–250	80–250
	$V_{5}$	<45%		
	$V_{10}$	<30%		
	$V_{20}$	<15%		
Right Lung	Mean dose	4Gy		
	$V_{5}$	<20%	120–250	120–250
	$V_{10}$	<0.5%		
TLV	Mean dose	6.5Gy		
	$V_{5}$	<25%	none	none
	$V_{20}$	<10%		
Right Breast	Mean dose	2.5Gy	120–250	120–250
	Maximum dose	10Gy		
	$V_{5}$	<5%		
	$V_{10}$	<0.5%		

Rx: prescription; TLV: total lung volume

### C. Arc angle arrangements

**Figure 1:**
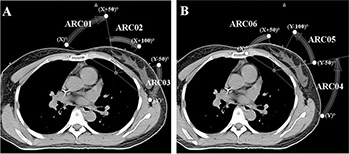
The arc angle arrangements for multiple partial volumetric modulated arc: (A) clockwise (CW) direction arcs (ARC01 to ARC03), (B) counter clockwise (CCW) direction arcs (ARC04 to ARC06). The angles of the gantry rotation for each arc are also indicated in this figure.

**Figure 2:**
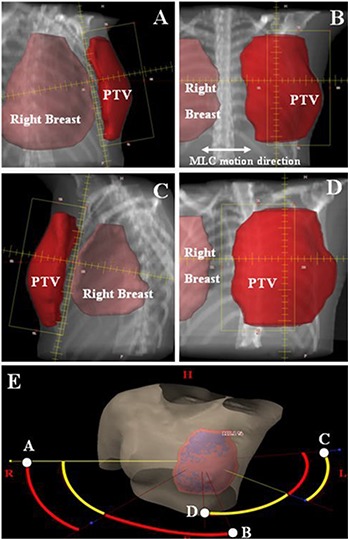
Examples of the field size opening on the beam's eye view (BEV): (A) the BEV on the start angle of ARC01, (B) the BEV on the start angle of ARC02, (C) the BEV on the start angle of ARC04, (D) the BEV on the stop angle of ARC05, and (E) the three‐dimensional display of six partial arcs. The CW arcs display in red curvature and the CCW arcs in yellow curvature. The corresponding BEVs of A to D are shown in this figure.

A MP‐VMAT plan consists of six partial arcs (ARC01 to ARC06), each with 50° gantry rotations. The directions of the ARC01 to ARC03 were clockwise (CW) and those of the ARC04 to ARC06 were counter clockwise (CCW), as depicted in Fig. 1. The start angle of ARC01 and stop angle of ARC03 were kept at the same angle as the conventional tangential technique. For example, we set the medial field gantry angle as X° and lateral field gantry angle as Y° in a left breast cancer case. The ARC01 arc starts from X° to (X+50)° followed by the ARC02 arc from (X+50)° to (X+100)° with different field size setting. The last CW arc (ARC03) starts with (Y−50)° and stops at Y°. For the CCW arcs, the ARC04 arc starts from Y° to (Y−50)° and is followed by the ARC05 arc, from (Y−50)° to (Y−100)° with different field size setting. The last CCW arc (ARC06) starts with (X+50)° and stops at X°. For ARC01, ARC03, ARC04 and ARC06, the collimator was rotated to 10°–12° and the jaw opening on the side near the chest wall was minimized to reduce the exposure to the left lung and the right breast, as shown in Fig. 2 (A) and (C). By incorporating these arc angles designs and the field size opening, we were able to incorporate the treatment with breath‐holding and also to minimize the exposure to the critical organs outside the PTV. To make sure the PTV was fully covered at the anterior end of the breast, the jaw opening on the side near the body surface is decided by the beam's eye view (BEV) arc animation. The ARC02 was designed to cover the lateral two‐thirds of the PTV, as seen in Fig. 2 (B), and the ARC05 to cover the medial two‐thirds of the PTV, as in Fig. 2 (D). The field size opening in the MLC motion direction varied as a result of the patient's breast shape and separation. The average field size opening among all the arcs and patients was 9.5 cm. (range 8.1 to 12.4 cm).

### D. Data Analysis

The plans were evaluated by using the dose‐volume histogram (DVH) of targets and critical organs. The percentages of a volume received at least m% of the prescription dose was expressed by $V_{m\%}$, and the dose to q% of the volume was expressed by $D_{q\%}$. The $V_{95\%}$, $V_{107\%}$, $D_{99\%}$, $D_{2\%}$, dose minimum and dose maximum of the PTV were reported. The target homogeneity index (HI)^(32)^ is expressed by ($D_{5\%}$–$D_{95\%}$) divided by the prescribed dose. The dose conformity to PTV is defined by the conformity index ${\left(\text{CI}\right)}^{\left(33\right)}95\%$ the ratio between the patient volume receiving at least 95% of the prescribed dose and the volume of PTV receiving at least 95% of the prescribed dose. The mean doses for all the relevant critical structures were assessed. The percentages of a volume received the dose of nGy were defined by $V_{n}$. For each plan, the following data were recorded: the $V_{5}$ and $V_{10}$ for all the critical organs; the $V_{25}$ and $V_{30}$ of the heart; the $V_{25}$ of the left ventricle; the $V_{20}$ for the left lung and the TLV; the $V_{5}$ and $V_{10}$ of the right breast. The $V_{5}$ and $V_{50}$ on the unspecified tissue were used to evaluate the other unspecified normal tissue area.

The treatment efficiency was evaluated in terms of the delivery time and the total MUs delivered. Six patient's MP‐VMAT plans were randomly selected and delivered on the phantom with a Varian iX linear accelerator. The delivery time for each arc was estimated by two approaches: (1) manual timer: two individuals recorded the start and stop times separately to include the delivery time plus the response time for the therapists; the two times were then averaged, and (2) machine timer: the time shown on the linear accelerator control monitor was recorded in minutes. One patient was used to compare the dosimetry of FIF with MP‐VMAT. The subfields of FIF were merged for the evaluation of treatment efficiency.

## III. RESULTS

### A. PTV dosimetry and the delivery efficiency

**Figure 3:**
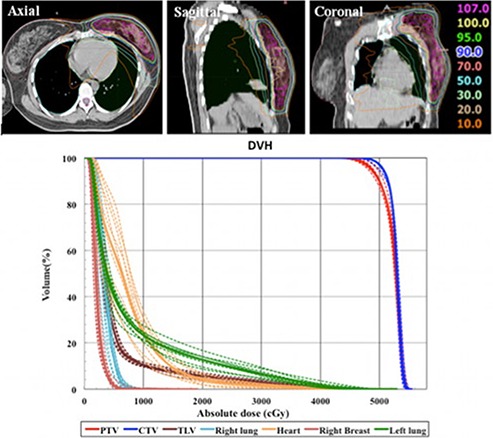
An example of the isodose distribution and sum dose‐volume histogram (DVH). The isodose distribution of axial, sagittal, coronal view for a patient, and the sum dose‐volume histogram (DVH) of the multiple partial volumetric‐modulated arc therapy in all left‐sided breast cancer cases. The dash lines show the DVH for all patients and the solid line indicate the average DVH for a specific organ or target.

Figure 3 shows the representative dose distribution in axial, sagittal, and the coronal views, and the sum DVH using the MP‐VMAT technique. The plan evaluation parameters for PTV and normal organs are listed in Table 2. The plans showed sufficient PTV coverage ($V_{95\%}$ >95%) in all cases, the maximum doses were within ±1% of 110% of the prescription dose, and the $V_{107}$ was 7.75% ±4.96%. The high degree of homogeneity and conformity was illustrated by the mean of HI as 10.6±0.99 and of CI as 1.21±0.03, respectively.

**Table 2:** The result of plan evaluation parameters for multiple partial VMAT plans.

*Structures*	*Range*	*Mean±SD*
PTV		
Minimal dose (%)	73.9–84.5	79.26±3.82
Maximum dose (%)	109.6–110.7	110.1±0.35
$V_{107}$ (%)	3.12–18.6	7.75±4.96
$D_{99\%}$ (Gy)	45.56–47.68	46.38±0.01
$D_{2\%}$ (Gy)	54.05–54.72	54.27±0.00
$V_{95\%}$ (%)	95.55–98.79	96.91±1.01
$\text{HI}-(D_{5\%}-D_{95\%})/\text{Rx}$	8.55–11.61	10.61±0.99
C$I_{95\%}$	1.13–1.24	1.21±0.03
Heart		
Mean dose (Gy)	5.55–9.55	7.61±1.38
$V_{5}$ (%)	35.57–81.60	59.73±15.87
$V_{10}$ (%)	13.02–33.81	24.39±6.82
$V_{25}$ (%)	0.69–4.10	2.52±1.11
$V_{30}$ (%)	0.37–2.43	1.57±0.71
Left Ventricle		
Mean dose (Gy)	5.51–10.85	7.32±1.49
Maximum dose (Gy)	85.3–104.00	93.23±6.62
$V_{25}$ (%)	1.63–7.99	5.15±2.23
Left Lung		
Mean dose (Gy)	7.60–9.30	8.22±0.57
$V_{5}$ (%)	31.62–44.24	40.46±3.81
$V_{10}$ (%)	20.39–27.43	23.32±2.07
$V_{20}$(%)	8.69–16.71	12.71±2.32
Right Lung		
Mean dose (Gy)	2.61–4.03	3.44±0.43
$V_{5}$ (%)	5.81–18.89	13.21±4.50
$V_{10}$ (%)	0.00–0.33	0.08±0.10
Total lung volume		
Mean dose (Gy)	5.08–6.02	5.57±0.36
$V_{5}$ (%)	20.28–31.04	25.39±3.88
$V_{20}$ (%)	3.90–7.06	5.66±0.89
Right breast		
Mean dose (Gy)	1.82–2.48	2.13±0.22
Maximum dose (Gy)	6.94–19.2	9.72±3.55
$V_{5}$ (%)	0.33–4.75	1.83±1.22
$V_{10}$ (%)	0.00–0.39	0.04±0.12
Unspecified tissue		
Mean dose	4.72–5.76	5.34±0.37
$V_{5}$ (%)	19.95–25.03	22.23±1.57
$V_{50}$ (%)	0.18–0.73	0.50±0.14

Rx: prescription; HI: homogeneity index; CI: conformity index.

**Figure 4:**
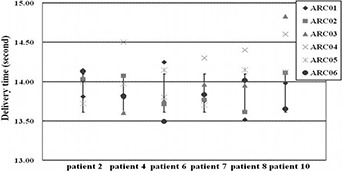
The time recorded from the manual timer approach, including the data from ARC01 through ARC06 with six randomly selected patients.

**Figure 5:**
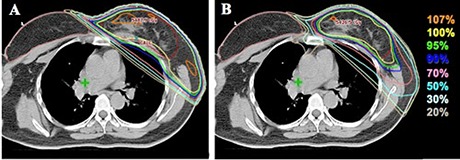
The isodose distribution for (A) field‐in‐field (FIF) and (B) multiple partial volumetric‐modulated arc therapy (MP‐VMAT) technique.

In terms of the delivery efficiency, the results from the manual timer for the delivery time of each arc versus each patient were plotted in Fig. 4. The mean of delivery time for each arc was 13.9 seconds (range 13.4–14.9 seconds) among six patients. The mean of delivery time recorded by the machine for each arc was 0.216 minutes (13.0 seconds), with a maximum of 0.23 minutes (13.8 seconds), and minimum of 0.21 minutes (12.6 seconds). The average MU among ten patients was 511 MUs (range 443 to 594 MUs). For FIF technique, the average MUs was 107±8 MUs and average delivery time was 14.7±2.0 seconds per field. The isodose curve comparison with FIF is shown in Fig. 5. The MP‐VMAT shows comparable isodose distribution with conventional FIF technique in the dose low area and greater conformity than the FIF. The $V_{107\%}$ of MP‐VMAT (5.22%) was less than FIF (11.5%) on the test patient.

### B. Normal tissue dose evaluations

Table 2 shows that the mean dose of the heart was 7.61±1.38 Gy; only one patient had a value of 9.55 Gy, slightly higher than the mean heart dose criteria. The $V_{5}$ of 59.73% ±15.87% and $V_{10}$ of 24.39% ±6.82%; 70% of patients met the criteria for the heart. In addition, the $V_{25}$ of 2.52% ±1.11% and $V_{30}$ of 1.57% ±0.71% for the heart showed superior organ sparing in the high‐dose region for all cases.

The mean dose for the left lung was 8.22±0.57 Gy, and the $V_{5}$, $V_{10}$ and $V_{20}$ was 40.46% ±3.81%, 23.32% ±2.07%, and 12.71% ±2.32%, respectively. Only one out of ten patients did not meet the $V_{20}$ constrain and had a value of 16.71%. The mean dose of right lung was 3.44±0.43 Gy below the constraint of mean lung dose < 4 Gy with only one exception, a patient who had a mean dose of 4.03 Gy. All the patients met the mean dose and the $V_{20}$ constrains for the TLV. The $V_{5}$ of the TLV was 25.39% ±3.88%, including three of the patients having values close to 30%.

The right breast had a mean dose of 2.13±0.22 Gy, $V_{5}$ of 1.83% ±1.22%, and $V_{10}$ of 0.04% ±0.12%. All the patients met the criteria for the right breast. The unspecified tissue had a mean dose of 5.34±0.37 Gy and $V_{5}$ of 22.23% ±1.57% in volume. The volume of the unspecified tissue receiving 50 Gy was 0.50% ±0.14%.

## IV. DISCUSSION

Compared to the advanced techniques, such as fixed‐beam IMRT and tomotherapy, the conventional tangential techniques (FIF or wedge) had doses more confined inside the radiation field and less low‐dose region ($V_{5}$) as described below. The mean integral dose is usually greater in these advanced techniques as a result of multiple beam directions passing through regions outside the PTV. The problem of low‐bath dose region and high integral dose was, at least partially, resolved in our MP‐VMAT strategies with the result of less low‐bath dose to the lung area, as shown in the axial view in Fig. 3. The high‐dose regions on the organ‐at‐risk were comparable or better than the other advanced techniques.

In addition, our findings show that the MP‐VMAT provides superior normal organ sparing with comparable integral dose to the conventional techniques, as described below. The MP‐VMAT provides advantages of conformity, dosimetry, and the hot spot area over FIF while the FIF may have the hot spots outside the target area, as shown in Fig. 5. Nevertheless, the FIF technique may provide shorter delivery time where it depends on the number of subfields and the MLC transition; it is a case‐by‐case scenario. The MP‐VMAT can serve as an alternative method to achieve both dosimetric and delivery time advantages while applying breathing control method.

### A. Radiation‐induced pneumonitis

The results of our study have shown significant dose sparing for left lung as compared to that reported by Popesecu et al.^(22)^ using the VMAT technique. A study reported by Kwa et al.^(34)^ that included 540 patients, found no radiation pneumonitis for 64 patients who received a mean lung dose up to 8 Gy, which is higher than the 5.57 Gy mean lung dose (Gy) by our MP‐VMAT design. The probability of developing Grade 2 radiation pneumonitis was low for patients who had less than 22% of normal lung volume irradiated with more than 20 Gy.^(35)^ The evaluation of TLV in our treatment design showed $V_{10}$ was significantly lower than the criteria reported by Graham et al.,^(35)^ and the $V_{5}$ was comparable to the FIF technique reported by Goddu et al.^(18)^ Based on these evaluation results on lungs, the likelihood of developing radiation‐induced pneumonitis by our MP‐VMAT design is extremely low.

### B. Cardiac toxicity

In this study, the heart mean dose (7.61±1.38 Gy) was much less than that of 12.2±1.8 Gy reported by Goddu et al.^(18)^ for tomotherapy, and 8.7–21.1 Gy for the best cases reported by Fogliata et al.^(13)^ for the IMRT technique. The mean heart dose of our MP‐VMAT design is comparable to the three‐dimensional FIF technique 7.5±3.4 Gy reported by Goddu et al.^(18)^ The $V_{5}$ of the heart shows large variations and this is possibly due to the CT images obtained in free breathing. The $V_{10}$ for the heart was 24.39% ±6.82% (range 13.02–33.81), which has superior organ sparing as compared to the 35.7% (range 28.7–42.5) using the VMAT technique, and is comparable to 24.1% (range 18.1–33.6) using the standard wedge technique reported by Popsecu et al.^(22)^ Although the inclusion of the internal mammary chain into the treatment region in some studies might significantly contribute the doses to the heart, our MP‐VMAT design provides good dose sparing for the heart.

Marks et al.^(6)^ observed that there were no significant cardiac perfusion defects if less than 5% of the left ventricle volume received 50% or more of the prescribed dose. The cardiac toxicity should be minimal if the $V_{25}$ of the left ventricle is not higher than 5.0%, which is close to the $V_{25}$ in our study. Therefore, minimal radiation‐induced cardiac toxicity is expected. If the deep inspiration breath‐hold technique is used, the irradiated cardiac volumes can be significantly reduced, and the dose to the heart will be further reduced.

### C. Secondary breast cancer

In a retrospective study on 41,109 breast cancer patients, Boice et al.^(11)^ found a relative risk of only 1.19 for developing secondary cancer on right‐sided breast cancer after breast irradiation. Our mean dose of 2.13±0.22 Gy to the contralateral breast was less than the 4.3±0.7 Gy reported by Goddu et al.^(18)^ and the 2.82 Gy by the Boice et al.^(11)^ Also, Johansen et al.^(12)^ had compared the risk of radiation‐induced malignancy in contralateral breast with IMRT and RapidArc; the study indicated a potentially higher risk could be associated to IMRT treatments with fixed gantry. In our study, the $V_{10}$ dose for the contralateral breast was much lower than the 0.7±0.8 (Gy) reported by Johansen et al. Therefore, the chance of developing secondary breast cancer on the contralateral breast should be minimal by using the MP‐VMAT design.

### D. Delivery efficiency

In our study, the maximum delivery time for a single arc was 14.9 seconds, and the average was 13.9 seconds by manual timers and 13.0 seconds by the machine timers. Wong et al.^(31)^ had investigated the breath‐holding technique using the ABC device on patients. The results shown with minimal duration of 15 seconds for one lung cancer patient and 20 seconds or more for all other patients. From above results, the delivery of each arc in the MP‐VMAT can be achieved within one breath‐holding. The treatment time for tomotherapy reported by Goddu et al.^(18)^ was an average of 14.6 minutes, and the IMRT technique reported by Popsecu et al.^(22)^ was an average of 8.8 minutes. In our study, the total delivery time on six partial arcs only took an average of 1.4 minutes for 1.8 Gy. Our MP‐VMAT technique not only significantly reduced the delivery time compared to other advanced techniques, but also preserved the major advantage of the VMAT technique for less delivery time. The efficient delivery without any interruption with MP‐VMAT arc arrangements can be achieved. However, a precaution should be noted: due to the short delivery time strategy for MP‐VMAT, the breath‐holding technique is suggested to maintain the patient in the same position to minimize dosimetric errors. For the clinical implementation, the patient position online verification can be verified using cine electronic portal imaging device (EPID) images as a quality assessment tool.

## V. CONCLUSIONS

Our MP‐VMAT technique for left‐sided breast cancer patients achieved adequate target dose coverage while maintaining acceptable low doses to organs‐at‐risk, and therefore reduced the potential for induction of second malignancy and side effects. The low‐dose bath area was much less than fixed‐beam IMRT or helical tomotherapy. The efficiency of treatment delivery could minimize the breathing effect, provide the patient with comfort, and achieve precise treatment with a breathing control system. The MP‐VMAT has been proven to be a reliable and feasible method in clinical practice.

## Acknowledgments

The authors would like to thank the physicist and physician group in Chang Gung Memorial Hospital‐Linkou who provided input for this study.
